# Phylodynamic analysis of a viral infection network

**DOI:** 10.3389/fmicb.2012.00278

**Published:** 2012-07-31

**Authors:** Teiichiro Shiino

**Affiliations:** Infectious Diseases Surveillance Center, National Institute of Infectious DiseasesTokyo, Japan

**Keywords:** phylodynamics, transmission network, phylogenetic inference, infection dynamics, scale-free network

## Abstract

Viral infections by sexual and droplet transmission routes typically spread through a complex host-to-host contact network. Clarifying the transmission network and epidemiological parameters affecting the variations and dynamics of a specific pathogen is a major issue in the control of infectious diseases. However, conventional methods such as interview and/or classical phylogenetic analysis of viral gene sequences have inherent limitations and often fail to detect infectious clusters and transmission connections. Recent improvements in computational environments now permit the analysis of large datasets. In addition, novel analytical methods have been developed that serve to infer the evolutionary dynamics of virus genetic diversity using sample date information and sequence data. This type of framework, termed “phylodynamics,” helps connect some of the missing links on viral transmission networks, which are often hard to detect by conventional methods of epidemiology. With sufficient number of sequences available, one can use this new inference method to estimate theoretical epidemiological parameters such as temporal distributions of the primary infection, fluctuation of the pathogen population size, basic reproductive number, and the mean time span of disease infectiousness. Transmission networks estimated by this framework often have the properties of a scale-free network, which are characteristic of infectious and social communication processes. Network analysis based on phylodynamics has alluded to various suggestions concerning the infection dynamics associated with a given community and/or risk behavior. In this review, I will summarize the current methods available for identifying the transmission network using phylogeny, and present an argument on the possibilities of applying the scale-free properties to these existing frameworks.

In their natural habitat, various pathogen groups exist in genetically and environmentally diverse human populations. Amongst the many infectious agents, droplet- or sexually transmitted viruses spread most rapidly through complex human networks. To control these types of viral diseases, we have to learn about the behavior of pathogens in relation to their host populations. Factors that influence and determine the incidence and distribution of infectious diseases have been investigated extensively in the field of epidemiology.

## Limitations of the classical methods for estimating transmission networks

Conventionally, epidemiological researchers have generally derived interpretations of the contact network by using interview or other measures available in the clinic. However, these methods have inherent issues in cases where the virus causes long-term chronic infections or short-term rapid transmissions. Diagnoses of infection cases of chronic viruses in early phase are usually made in only a small population of individuals (Pao et al., 2005; Pilcher et al., 2005). Clinical surveillance can also acquire a small number of patients as compared with a whole population of person who is infected in virus with very rapid spreading, such as pandemic influenza. Therefore, the clinic-based analyses of viral epidemiology have been restricted to low-density samples and this factor may cause a bias in the results towards under-reporting of infection networks or “clusters” (Brown et al., 1997; Lewis et al., 2008). One solution to detect these infection clusters in virus transmission networks is a phylogenetic analysis of population-based samples of viral genetic sequences. A number of studies have identified the clusters by elucidating the evolutionary relationship of human immunodeficiency virus (HIV) (Salminen et al., 1993; Brown et al., 1997; Yirrell et al., 1997), hepatitis C virus (HCV) (Aitken et al., 2004) and the influenza virus (Nelson et al., 2007; Nelson and Holmes, 2007). Nevertheless, in the phylogeny that exhibits a star-like divergence pattern, the analysis using sequences from population survey can only provide limited evidence for the cluster. Sexually-transmitted populations of HIV-1 are representative of diseases showing this type of divergence patterns. For example, a population-based phylogenetic analysis of HIV-1 in the UK (Brown et al., 1997) could only identify a limited number of clusters even though the primary infected individuals were recruited from a cohort. A similar study conducted in Quebec (Brenner et al., 2007) showed to a certain extent evidence for the cluster, however most of the findings were gathered from intravenous drug users or from the sexual transmission patterns of a men who has sex with men (MSM). These major risk factors are still relatively unexplored and therefore poorly elucidated. These challenges are mostly due to the fact that most of the patients recruited were in the phase of chronic infection, despite the fact that in both of these research studies the recently (<6 month) seroconverted individuals were recruited from cohorts. Because a diagnosis of acute infection is usually made in only a small proportion of individuals with HIV-1 (Pilcher et al., 2005), the samples will inevitably contain viruses at chronic infection phase. Moreover, multiple introductions into target populations (Korber et al., 2000) will result in higher diversity amongst each virus group. In these situations, simple phylogenetic analyses that only employ sequences of virus at the chronic infection phase will generate inaccurate outputs due to computational bias, and skew the results thus underestimating the number of clusters (Brown et al., 1997). To resolve this supposed bias, it is necessary to obtain a larger number of sequences for analysis and/or use more sophisticated methods to infer evolutionary relationships from the sequences.

Contact tracing by interview data, which plays a key role in establishing the etiology of some infectious diseases (Klovdahl, 1985), may be difficult to carry out at the sites of epidemics. Transmission networks reconstructed by phylogenetic analysis have been considered as the standard host contact network in many studies. However, results gained by using both of the current methods have often been inconsistent due to long-term infections, a low average risk of transmission, and a relatively high rate of exposure to the virus (Wawer et al., 2005). The contact tracing method cannot effectively identify the specific instances of contact detected in the interviews associated with the infection, while the conventional sequence-based analysis cannot sufficiently confirm the results to provide quantitative descriptions concerning the transmission networks due to the above mentioned reasons.

These difficulties could potentially be overcome by acquiring an adequate number of viral sequences and also by an improved method for estimating the divergence time for each phylogenetic node. A number of recent advances in clinical and computational science have introduced the possibility of developing a novel more efficacious approach. Rapid developments in DNA sequencing technology have catalyzed the advent of medical diagnostics using viral genome sequences. In particular, genotype-based resistance tests are commonplace in anti-viral therapeutics for patients infected with HIV, HBV or HCV. Progress made in computational technologies has also facilitated large-scale sequence analysis of clinical diagnostic data. With advancements in evolutionary biology, the evolutionary dynamics of a population can now be inferred from sequence data and incorporated with sampling dates. Such evolutionary dynamics information of a pathogen derived from these analyses can then be combined with epidemiological data to illustrate the influences of host transmission dynamics, immunity, and treatments against specific genetic variations of pathogen. Such a series of analyses is now referred to as “phylodynamics” (Grenfell et al., 2004; Holmes and Grenfell, 2009). Phylodynamic frameworks require sufficient sequence diversities of the sample dataset with respect to spatial as well as temporal variations. Thus, RNA viruses, which have high substitution rate, high growth rate, and a short generation time, are especially advantageous and amenable to investigation (Grenfell et al., 2004; Kühnert et al., 2011).

## Seeking transmission networks using phylodynamics

Identifying transmission networks is one major issue in the epidemiological analysis of infectious diseases. In research performed on HIV sexual transmission networks, drug resistance tests accompanied by HARRT have helped to provide a sufficient number of sequences. Even in other viral diseases, the sequence datasets accepted in the framework may be available under an arrangement of surveillance system with the sequence database. The divergence time, another piece of the framework, has been estimated from the time of the most recent common ancestors (tMRCAs) for each node of phylogeny, and dating of phylogenetic tree, including tMRCA estimation, is one of the recent achievements of modern theoretical biology (Drummond et al., 2005, 2006). Currently, phylogenetic inference using Bayesian coalescent Markov Monte Carlo (Bayesian MCMC) method (Pybus et al., 2003; Drummond and Rambaut, 2007) is commonly performed in this step. It is well-known that due to their high mutation and proliferation rates, the sequence evolution of an RNA virus occurs on a time scale when any public health measures are being conducted, suggesting that a time-based phylogenetic inference, which usually requires samples taken in extremely different ages such as fossil-derived PCR sequence, is easily applicable to RNA viral sequences (Pybus et al., 2003). An excessive number of sequences would increases the probability of acquisition of the transmission networks in a phylogeny, and a relative date of infection estimated by each divergence time in a phylogeny provides the missing links for contact cases.

**Figure 1:**
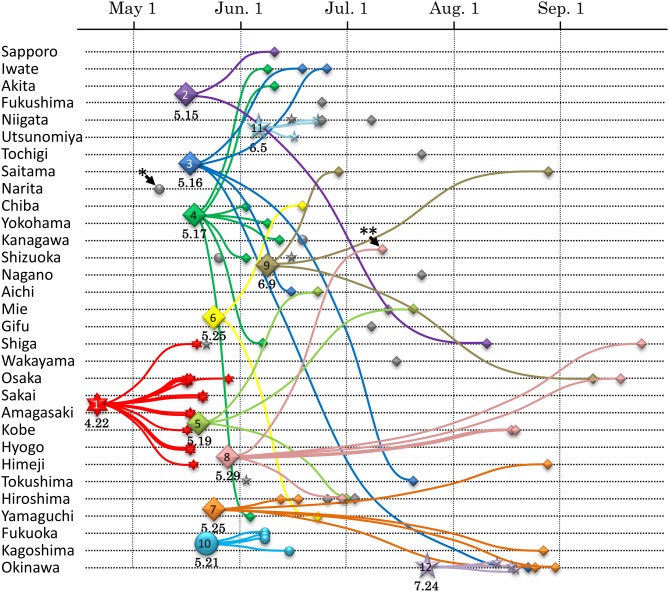
**Transmission dynamics of A(H1N1)pdm epidemic in Japan estimated by phylodynamics.** The vertical and horizontal axes show geographic localities and the times of virus collection, respectively. Chronological phylogeny was inferred by BEAST v1.5.4 using the general time reversible model, taking into account site heterogeneity and invariant sites (GTR+I+G) and the logistic population model (Drummond and Rambaut, 2007). Seventy-five isolates of A(H1N1)pdm viruses from Japan are plotted with the small symbols. The MRCA of each transmission network inferred from the phylodynamics are plotted with the large symbols. The numbers below the large symbols display the tMRCA date. This figure is cited from Figure 4 in our previous report (Shiino et al., 2010).

Quantitative description of a transmission network using phylodynamics represents a temporal and spatial profile for certain viral epidemics. For example, intra-national epidemiological study of pandemic influenza A(H1N1) in 2009 was analyzed by this framework (Shiino et al., 2010). In this study, an endemic transmission network detected in the phylogeny was regarded as a single transport case of A(H1N1) virus in Japan. The local spreading profile of 12 cases was illustrated by the date (when the case was introduced) and locality (how the virus was spread) of the infection (Figure 1). This type of approach is performed in a more detailed manner on sexual-HIV epidemics in the UK using partial *pol* regions from over 10,000 patients (Lewis et al., 2008; Hughes et al., 2009). A large majority of MSM persons were linked to more than one other individual and 25% engendered large transmission networks (Lewis et al., 2008), whereas only 5% of individuals generated large clusters in heterosexual transmissions (Hughes et al., 2009). On the issue of transmission intervals estimated from tMRCA, the median interval for by MSM [i.e., 13 months; (Lewis et al., 2008)] was less than half of that estimated for heterosexual individuals [i.e., 27 months; (Hughes et al., 2009)]. Thus, the phylodynamics revealed an aspect of a viral spreading pattern that was associated with a given community or risk behavior that had not been previously elucidated.

## Estimating epidemiological parameters from sequence diversity

**Figure 2:**
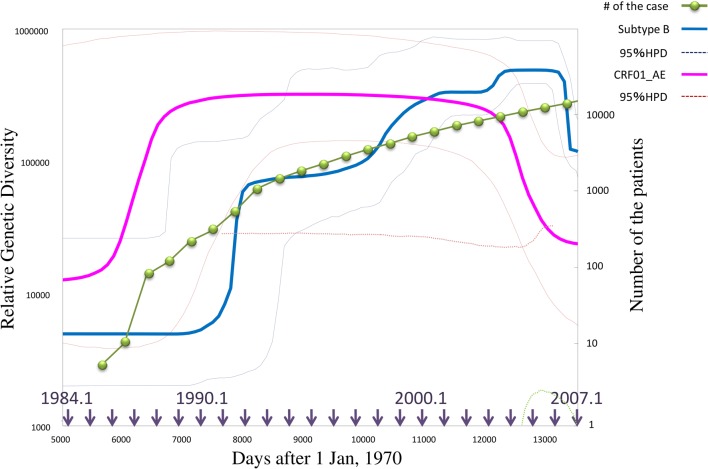
**Population dynamics inferred by Bayesian skyline plot analysis of HIV-1 epidemics in Japan.** This graph represents the median estimate of the genetic diversities, i.e., relative population sizes, of HIV-1 Subtype B (blue line) and CRF01_AE (pink line) in Japan, with 95% posterior intervals (dotted line) estimated by BSP. The green line shows the number of HIV-1 infection cases in Japan. The horizontal axis indicates days after the first day of 1970 with the arrows showing approximate date. BSP is performed using BEAST v.1.5.4 with GTR+I+G (Drummond and Rambaut, 2007), 50 million states of MCMC runs, and burn-in of 4 million states. The sequence dataset was provided by the research program of Japanese Drug Resistance HIV-1 Surveillance Network Group (Gatanaga et al., 2007; Hattori et al., 2010) making use of over 2000 protease-RT sequences from Japan.

Another area of interest for epidemiologists is the estimation of parameters affecting the dynamics of a particular pathogen. Predicting the population dynamics of a pathogen requires precious quantification of the key parameters in the population model. Although generally, it has been derived from enumeration of data on the disease incidence, parameters inferred by phylodynamics are also applicable in describing complex population dynamics of an RNA virus. When a sequence variation in a gene population primarily depends on the neutral mutation, the expected value of mean nucleotide diversity is proportional to N_e_υ, where N_e_ indicates an effective population size and υ signifies the total mutation rate per loci (Kimura, 1969). Since υ is uniform for long time-scales (although the generation time of the transmitted virus may fluctuate), the observed nucleotide diversity is thought to be a relative size of the viral population (Drummond et al., 2005). The coalescent tree analysis in a phylodynamic framework allows measurement of the nucleotide diversity for each time unit from a time-slice of the chronological phylogeny. The consequence of this slicing is the Bayesian skyline plot (BSP), which represents a piecewise graphical demonstration of population dynamics of the virus (Drummond et al., 2005). Note that the BSP does not demonstrate population dynamics of the host individual but rather for the pathogen itself, although both are consistent in the case of a fixed number of transmitted viruses to the host. As shown in Figure 2, the BSP can illustrate a feature of the epidemic along with a temporal component (Rambaut et al., 2008). Moreover, the BSP is useful in analyzing the intra-host virus struggle against an immune response (Bernini et al., 2011). Additionally, better precise estimates of the parameters are now capable of improved assessment using the phylodynamic framework. The population growth rate (*r*) can be estimated by the Bayesian MCMC inference (Drummond and Rambaut, 2007) as well as the maximum likelihood phylogeny with branch length correction for the sampling date (Pybus and Rambaut, 2002). The mean time of infectiousness (*D*) is dependent on a function of distribution of the generation time periods that elapsed between transmission processes [*w*(*t*)] (Grassly and Fraser, 2008), although this can also be inferred from the phylogenies, it is difficult to determine one estimates due to the various properties of viral infections (i.e., fluctuating viral load, and the wide range of transmission probability with respect to risk behavior) (Sherlock, 1993; Chevaliez and Pawlotsky, 2007; Romano et al., 2010). When we have obtained *r* and *D*, we can approximately estimate the basic reproductive number (*R*_0_) using the relation *R*_0_ = 1 + *rD* (Pybus et al., 2001). Estimating *r* and *R*_0_ in HCV-infected individuals revealed that subtype 1b, which is found chiefly amongst elderly individuals with a history of blood transfusions, spreads slower than the compared to other subtypes (Romano et al., 2010).

## Random graph analysis of viral transmission dynamics and phylodynamics

**Figure 3:**
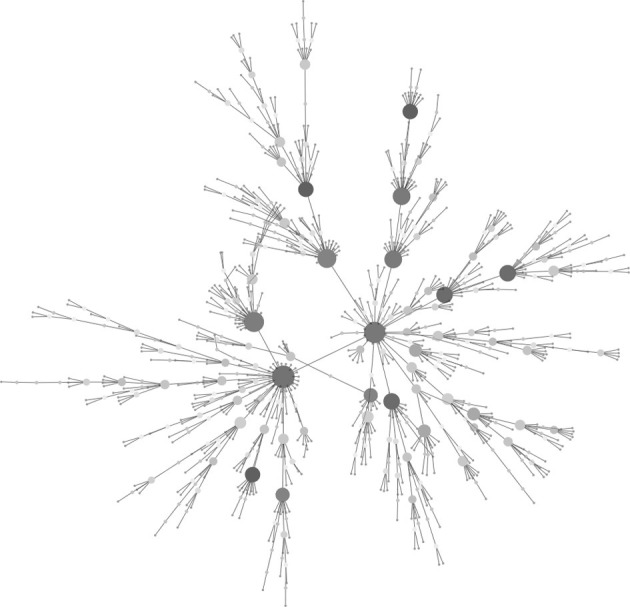
**Example of a scale-free network.** The graph consisting of 1000 nodes was generated with the Barabási and Albert model (Barabási and Albert, 1999). Large and deeper-colored nodes show higher connectivity. Note the majority of nodes have few connections. The graph was generated by igraph 0.6 using ba-model and visualized in Cytoscape 2.5.

**Figure 4:**
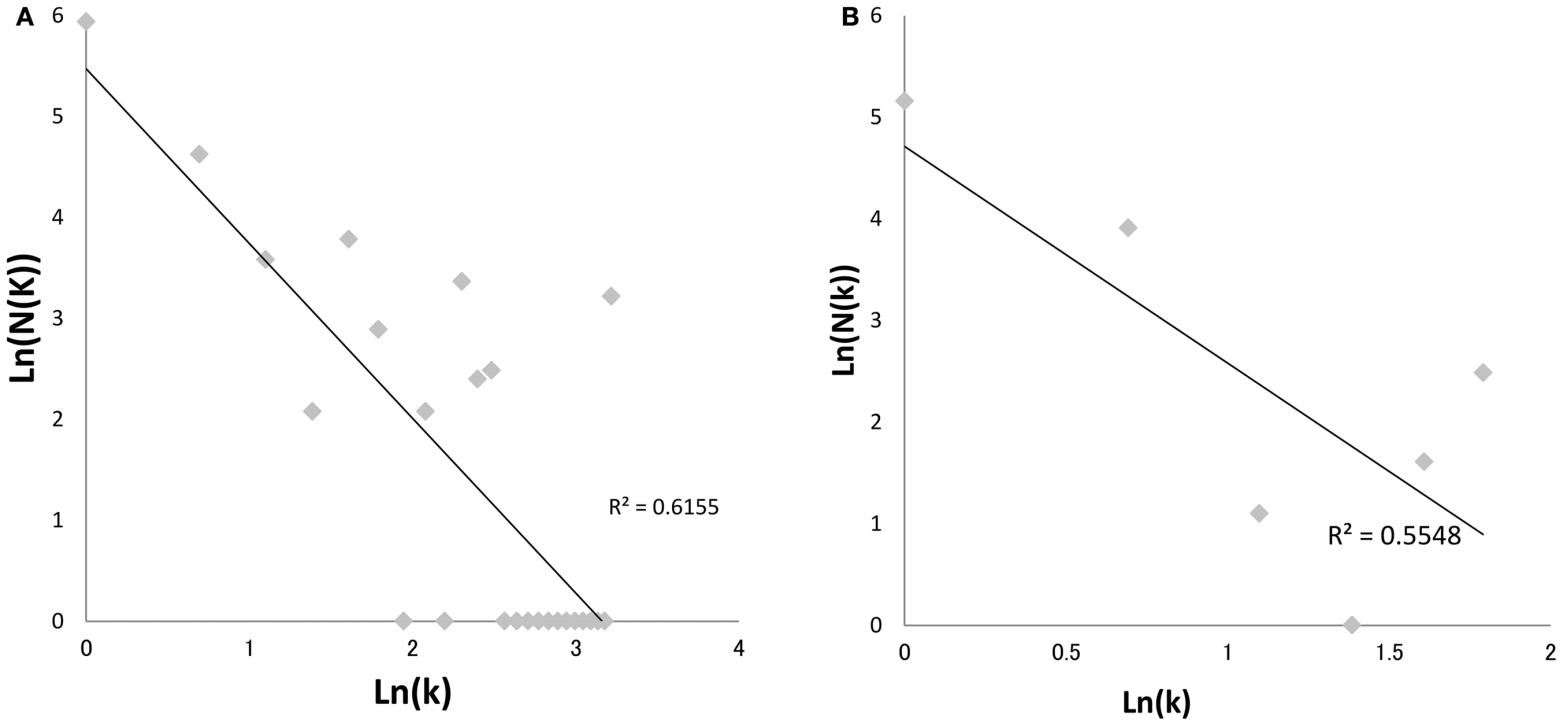
**Power-law plot of HIV-1 transmission network in Japan.** The graphs depict log-log plots of the numbers of patients with k contacts [*N*(*k*)] against the number of contacts (*k*) in HIV-1 clusters of Japan subtype B **(A)** and CRF01_AE **(B)**. Regression line was calculated using the least-square method. The adjusted coefficients of determination (*R*^2^) were 0.5548 (*p* = 0.04) and 0.6155 (*p* < 0.01), respectively. The Neighbor-Joining trees were reconstructed using MEGA version 5.0.5 (Tamura et al., 2011), and >80% bootstrap probabilities adhering to the criteria in a previous report (Shiino et al., 2010) were adopted in the significant clades. The statistic analyses were calculated using R version 2.10.0. The sequence dataset was provided by the research program of Japanese Drug Resistance HIV-1 Surveillance Network Group (Gatanaga et al., 2007; Hattori et al., 2010) using over 2000 protease-RT sequences from Japan.

Since Watts and Strogatz (1998), Barabási and Albert (1999), introduced the “small-world” and “scale-free” network models (Figure 3) to random graph theorem, respectively, it have been elucidated that the scale-free and small-world properties are observed in many human-intervened communicative networks such as the internet and infectious diseases. Pastor-Satorras and Vespignani (2001) demonstrated that computer viruses rampant on the internet spread through a scale-free network, which can drive the viruses to spread even when infection probabilities are negligibly small. This prediction can be applied to not only computer systems but also human pathogenic viruses. Scale-free properties are observed in transmission networks reconstructed by the phylodynamic framework, e.g., connectivities of the individuals in the network often follow a power law (Figure 4). Such features have been found in the phylodynamic network of HIV both in MSM (Lewis et al., 2008) and heterosexual populations (Hughes et al., 2009) and in HCV groups (Romano et al., 2010). Moreover, the transmission network for the HIV epidemic among MSM in the UK was recently reanalyzed using fine distribution model with the preferential attachment process. Observed distribution specifically fitted to the Waring distribution at all time-depths of the phylodynamic clusters (Leigh Brown et al., 2011). These findings give a significant message for preparation of a public health measures; Lloyd et al. stated in their perspective in Science (Lloyd and May, 2001) that “the study highlights the potential importance of studies on communication and other network, especially those with scale-free and small world properties, for those seeking to manage epidemics within human and other animal population.” Under the scale-free and small-world condition, an infection will spread regardless of its transmissibility and a control program targeted at highly connectable individuals (i.e., super-spreaders) is important to curb the epidemic (Keeling and Eames, 2005). A phylodynamic framework would help to decide a target population for the treatment and allow a decrease in the cost of treatment.

## Identification of the transmission network using the scale-free feature

**Figure 5:**
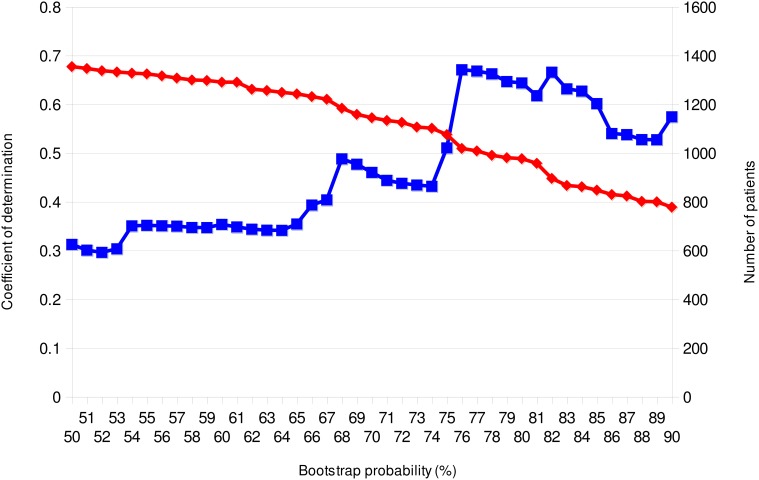
**Bootstrap-scanning on power-law fitting of clusters in the Neighbor-Joining tree.** The adjusted coefficients of linear regression determination of log-log plots and the number of individuals in the clusters are plotted on the bootstrap cut-off probability for the clade distinction. The blue line with square symbols and the red line with lozenge symbols show the coefficients of determination and the numbers of individuals included among the clusters, respectively. The phylogenies were reconstructed using MEGA version 5.0.5 (Tamura et al., 2011). MEGA output was analyzed using a combination of PERL and R scripts. This analysis was performed using 1882 sequences of the protease-RT regions of HIV-1 Subtype B in Japan; the data was provided by the research program of Japanese Drug Resistance HIV-1 Surveillance Network Group (Gatanaga et al., 2007; Hattori et al., 2010).

From previous discussions, it is evident that phylodynamics can engender important knowledge about the epidemiology of infectious diseases. The problem is that no accurate and consistent process for deciding the transmission network on the phylogeny is present in this framework. Before conducting Bayesian MCMC analysis, which is a core process in phylodynamics, one has to identify transmission networks from monophyletic groups (clade) inferred by the conventional phylogenetic tree analysis. It is difficult to evaluate the credibility of the clade in the pathogen phylogeny, especially in star-like trees observed in chronic (e.g., HIV-1) and/or pandemic (e.g., novel influenza) infections. While it is common to use the bootstrap probability method for verifying the reliability of the clade, this is a dubious index in the case of viral sequence analyses since it may be different from the probability distribution of error when it is applied to the viral sequences that widely fluctuate their base substitution rate along with the site or host environment. Although the posterior probability of nodes calculated in the Bayesian tree inference was sometimes used as phylogenetic support for each clade (Lewis et al., 2008), this method has a computational issue, as the MCMC search with large datasets requires a huge resource of computational power. In the case of imported infectious disease, robustness of the cladding is also examined by supplying a large number of closely related reference sequences (Hughes et al., 2009; Shiino et al., 2010). However, this type of method depends on whether these reference sequences are freely available or otherwise. Consequently, here I wish to propose a method considered to be more effective in determining the cutoff value of bootstrap probability for transmission network reconstruction. As mentioned above, assuming that nearly all viruses transmitted along with the scale-free network, degree distribution of the number of members in valid networks estimated from the clades in a phylogeny should follow the power law. Therefore, if the relationship between the results of each bootstrap value of the observed phylogenetic cluster and fitting of the degree distribution to power law plot is investigated, a bootstrap probability for selecting the network to be adopted may be clear. Figure 5 showed the relationship between bootstrap probability of the neighbor-joining tree and the coefficient of determination (*R*^2^) in linear regression of log-log plots of the member distribution of the significant clades, using 1882 sequences of the pol domain of HIV-1 subtype B in Japan collected by the Japanese Drug Resistance HIV-1 Surveillance Network Group (Hattori et al., 2010). The *R*^2^ with regard to the power law fitting shows highest values at bootstrap probabilities between 76 and 82%, and decreased in both higher and lower the probabilities. On the other hand, the number of infected persons included in the significant clades decreased consistently as the bootstrap probability increased. This result suggests that the optimal bootstrap probabilities for the scale-free property, which is approximately 80% in this case, is present in a viral sequence data.

## Conclusion

In order to manage and control infectious diseases, it is important for epidemiologists to monitor and comprehensively analyze representative epidemiological indices. The phylodynamic framework proposed here can serve as a powerful tool for handling such data by integrating related areas of epidemiology such as population dynamics, genetics and molecular evolutionary research. Additionally, this framework is relevant with respect to aspects of pathogen evolution against immunological responses, drug administration and pathological development. Remaining challenges pivotal in the eventual success of the framework include necessary improvements in data sampling for both disease incidence and pathogenic sequences. At present, these types of data are only collected periodically. In addition, public sequence databases such as GenBank should begin to stringently record the collection date, location, and the accompanying clinical information. Achieving these objectives will allow the phylodynamic framework to future contribute to successful disease control.

### Conflict of interest statement

The author declares that the research was conducted in the absence of any commercial or financial relationships that could be construed as a potential conflict of interest.
